# Inflammatory mediators in polycystic ovary syndrome: the case of interleukin-18

**DOI:** 10.20945/2359-3997000000455

**Published:** 2022-01-01

**Affiliations:** 1 Hospital de Clínicas de Porto Alegre Unidade de Endocrinologia Ginecológica Porto Alegre RS Brasil Unidade de Endocrinologia Ginecológica, Serviço de Endocrinologia, Hospital de Clínicas de Porto Alegre (HCPA), Porto Alegre, RS, Brasil; 2 Universidade Federal do Rio Grande do Sul Departamento de Fisiologia Porto Alegre RS Brasil Departamento de Fisiologia, Universidade Federal do Rio Grande do Sul (UFRGS), Porto Alegre, RS, Brasil

Polycystic ovary syndrome (PCOS) is a multifactorial condition, characterized by clinical or biochemical hyperandrogenism, ovarian dysfunction, and/or polycystic ovaries. Insulin resistance and central adiposity are often present, and women with PCOS are at higher risk for metabolic comorbidities, such as dyslipidemia, prediabetes, and type 2 diabetes (1).

Current evidence suggests that women with PCOS have increased serum pro-inflammatory markers when compared to healthy controls, contributing to a chronic low-grade inflammatory state (2). Indeed, major pro-inflammatory cytokines, such as IL-6 and TNFα, may be derived from dysfunctional adipose tissue in PCOS and primarily secreted by resident macrophages (1). IL-6 induces the hepatic production of CRP, and women with PCOS exhibit elevated CRP levels, which are correlated with obesity and insulin resistance (3). Conversely, some other inflammatory mediators are under investigation in the context of their possible pathophysiological role on the chronic low-grade inflammatory state associated to PCOS. One of these biomarkers of inflammation that are under evaluation is interleukin-18 (IL-18).

IL-18, a pro-inflammatory cytokine, was first described as an interferon-gamma inducing factor (4) and has been related to distinct low-grade inflammatory conditions (5-7). Interestingly, besides being a marker of insulin resistance and cardiovascular disease, IL-18 have been linked to ovulatory dysfunction in PCOS (8). IL-18 in follicular fluid was also reported to be correlated with the response to ovarian stimulation, and successful pregnancy after IVF treatment (9).

In this issue of *AE&M*, Kabakchieva and cols. (10) report the associations between IL-18 and obesity, central adiposity, insulin resistance, and hyperandrogenism in a sample of women with PCOS. While women with and without PCOS presented similar IL-18 serum levels globally, when stratified by the BMI, participants from both PCOS and control group considered as overweight/obesity had higher IL-18 levels than the eutrophic participants. In addition, in paralel analyses, women with PCOS and insulin resistance, high waist-to-height ratio (WHtR) and high free androgen index (FAI) had higher levels of IL-18 compared to the corresponding opposite strata. In multiple linear regression, age, waist circumference, and fasting insulin were factors independently related to IL-18. The results of this study, analyzing IL-18 levels associated to specific PCOS-related factors, namely central obesity, insulin resistance and androgen excess, support the relevance of the anthropometric and hormone profile on the expression of a proinflammatory state. In this context, authors successfully chose an accurate adiposity index, the WHtR (11,12).

This study of Kabakchieva and cols. (10) also expands previous published data. In fact, as mentioned by the authors, previous studies have shown no consensual results, some presenting increased IL-18 levels in women with PCOS independently from obesity and others indicating that IL-18 circulating levels were impacted by obesity or insulin resistance. However, most of these papers were observational studies, with small sample sizes. Therefore, further prospective studies, with larger sample sizes and distinct ethnicities are warranted. Importantly, as lifestyle changes associated or not with pharmacological interventions are recommended for reducing metabolic risks in women with PCOS (13,14), the effect of interventional studies on the inflammatory status, by improving insulin sensitivity and/or suppressing androgen excess, might also shed light on this issue. In this sense, a recent meta-analysis including 27 studies has shown that the use of combined oral contraceptives was associated with a reduction of most inflammatory markers (15).

Considering the heterogeneous clinical presentation of PCOS, an individualized medical approach is consensual in managing PCOS, according to the presence of cardiometabolic risk factors, as well as the main complains of the patient, such as clinical hyperandrogenism, menstrual disorders or infertility. In contrast, from a research point of view, identifying whether IL-18, an inflammatory mediator, represents a reliable sign of metabolic alteration specific to PCOS, is still a challenge.
